# Strategic Use of Negative Emojis in Messaging-Based Interventions for Public Health Communication on Social Media: Mixed Methods Study

**DOI:** 10.2196/78824

**Published:** 2026-07-31

**Authors:** Yue Luo, Shubin Yu

**Affiliations:** 1 Department of Visual Communication Design, College of Fine Arts, Guangdong Polytechnic Normal University, Guangzhou, China; 2 Department of Communication and Culture, BI Norwegian Business School, Nydalsveien 37, Oslo, Norway, 47 41228055

**Keywords:** emojis, preventive behavioral intention, emotional valence, congruence, mobile health communication, social media engagement

## Abstract

**Background:**

Despite the growing importance of social media in mobile health (mHealth) communication, we lack a clear understanding of how emotional elements like emojis shape message effectiveness. Furthermore, since emojis are inherently tied to text, their impact may be highly dependent on the relevance and context of the accompanying written content.

**Objective:**

This study aimed to investigate how the valence of emojis affects the effectiveness of mHealth messages and to determine the role of emoji-text congruence in shaping message outcomes.

**Methods:**

A mixed-method approach was used, encompassing 3 complementary studies. First, an analysis of real-world health-related tweets (N_1_=257,648) quantified social media engagement in relation to positive, negative, and absent emojis. Building on these insights, 2 controlled experiments (N_2_=220; N_3_=190) further explored how negative emojis influence preventative health behaviors under varying levels of text congruence.

**Results:**

The automatic content analysis revealed that messages containing negative emojis generated significantly higher social media engagement than those with positive emojis (*β*=0.24, SE=0.03, *t*_257, 593_=9.59; *P*<.001). Subsequently, experimental findings indicated that negative emojis can effectively promote preventative health behaviors (*t*_218_=−4.15; *P*<.001). However, messages with high negative emoji-text congruence are more persuasive in promoting preventive behavior than those with low congruence (*F*_1, 186_=5.46, *η^2^*=.028; *P*<.05), highlighting the complex interplay between emotional signaling and message consistency.

**Conclusions:**

These findings advance our theoretical understanding of emoji-based health communication and provide practical guidelines for public health organizations seeking to optimize their mHealth messaging strategies. Our results highlight the need for careful consideration of both emotional valence and message coherence when designing health communications in the digital age.

## Introduction

### Background

In mobile health (mHealth), disseminating accurate, comprehensible, and persuasive health information is crucial [1]. Campaigns, whether in the form of health posts or advertisements, aim to foster positive attitudinal and behavioral shifts, which is especially important when conveying protective measures against infectious diseases such as Ebola, COVID-19, and Monkeypox, or promoting routine practices like physical examinations [1]. Given the vast reach and interactive nature of social media, public health advocates increasingly leverage these platforms to communicate vital information [2-5]. Within these digital interactions, emojis have become ubiquitous, functioning as paralinguistic cues that supplement textual content and amplify emotional expression [6-8]. Scholarly attention has thus turned to understanding how emojis support messaging-based interventions in mHealth, particularly since research indicates that their inclusion can evoke more intense emotional responses compared to text alone, potentially enhancing behavioral intentions [9-17].

While several studies have demonstrated that the presence of emojis generally benefits health communication [13,16,18,19], most have focused on their mere presence versus absence, without delving into the nuanced effects of specific emoji characteristics. Emojis are not monolithic; they vary in emotional valence and serve as potent social cues in online interactions [6]. In the context of health communication, positive emojis (eg,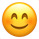, 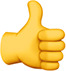) are conceptually defined as symbols that convey approach-oriented or supportive emotions, signaling safety or encouragement to cope [15,20]. By contrast, negative emojis (eg,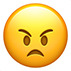, 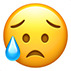) express avoidance-oriented, alarming, or aversive emotions, signaling risk or concern [16,17]. The emotions as social information (EASI) model provides a foundational framework by suggesting that emotional expressions influence behavior via two main pathways: a direct affective reaction (eg, emotional contagion) and a cognitive inferential process (eg, deducing meaning or intent) [21]. Despite its strengths, scholars call for more comprehensive testing of the EASI model across digital contexts to better understand the specific persuasive impact of emoji valence in health messaging [6,21]. A clear grasp of how emoji valence operates is essential for designing more effective public health interventions.

Beyond shaping attitudes and behavioral intentions, a cornerstone of health communication efficacy [22-24], the success of health messages on social media is reflected in engagement metrics. Social media engagement is the extent to which users interact with online content through behaviors such as liking, commenting, and sharing [25-27]. These metrics indicate that a message has captured audience interest, stimulated discussion, and achieved widespread dissemination. Moreover, the congruence between a message’s components—specifically, the emotional tone of an emoji and the accompanying text—is a known predictor of message credibility and persuasiveness [28,29]. Incongruence between emoji valence and textual content could compromise message clarity and reduce persuasive effectiveness.

This study addresses these gaps by examining the strategic use of negative emojis in public health communication through messaging-based interventions on social media. We integrate three complementary theoretical perspectives: the EASI model [21], which explains how emotional expressions shape behavior; emotional contagion theory [30], which details how negative emotions can rapidly spread within social networks; and schema-congruity theory [31,32], which elucidates how congruence between emojis and text facilitates multimodal cognitive processing. Guided by these theoretical underpinnings, we address the following hypotheses:

### Effectiveness of Emoji Valence on Social Media Engagement

The affective pathway of the EASI model posits that observed emotions can induce similar feelings in recipients, while Emotional Contagion Theory [30] indicates that negative emotions tend to spread more rapidly, serving as potent threat signals [33,34]. Previous research has shown that negative emotional cues, even when conveyed through emojis, can heighten risk perception and, in turn, drive user engagement as audiences seek, share, or discuss the information [35-37]. In the context of health communication, negative emojis act as digital “alarm signals,” motivating engagement through two complementary processes: (1) individual-level contagion (unconscious adoption of fear or concern) [30] and (2) social-level diffusion (sharing messages to alert others) [38]. Critically, negative emojis exhibit stronger contagion effects than positive ones.

Hypothesis 1: compared to positive emojis, using negative emojis in health-related social media posts will generate higher social media engagement.

### Effectiveness of Emoji Valence on Preventive Behavioral Intention

Previous studies have leveraged emoji valence to inform individuals’ perceptions and behaviors, such as emotional responses to food [39] and intentions to receive vaccination [16]. Preventive behavioral intention refers to the deliberate motivation to adopt health-protective actions and serves as a critical precursor to actual health behavior change [16,40-42]. Persuasive messaging often leverages emotional valence to amplify risk perception and motivate action [22,43]. Negative emojis (eg, 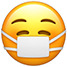, 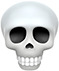) serve as condensed emotional cues that trigger a “risk-as-feelings” response, making abstract health risks more tangible and activating defensive motivations [43]. This process is consistent with the affective pathway anticipated by the EASI model and the human predisposition to give priority to negative signals as survival-relevant warnings [33,35].

Hypothesis 2: Compared to positive emojis, using negative emojis in health-related social media posts will increase individuals’ preventive behavioral intentions.

### Moderating Role of Emoji-Text Congruence on Preventive Behavioral Intention

In messaging-based health interventions on social media, emojis are often conveyed to users alongside textual messages. Schema-congruity theory [31,32,44] suggests that messages aligning with an individual’s existing schemas are processed more smoothly. People tend to have more positive attitudes toward messages that exhibit high internal congruity [32,44]. For example, in high-risk contexts, recommendations are more effective when the visual content, textual message, and information source are mutually consistent [1,16]. Erle et al [6] assert that although emojis enhance the comprehensibility of information on social media, this effect is not realized solely through perceived valence. According to the EASI model’s inferential pathway, the alignment between the emotional tone of emojis and the textual narrative builds a cohesive communicative schema. Negative emojis can enhance preventive intentions when they are congruent with risk-oriented text, whereas positive emojis may backfire when they are perceived as incongruent with the seriousness of the message. Although some degree of incongruence may introduce novelty effects [45,46], in health communication, a mismatch (eg, pairing positive emojis with severe warnings or negative emojis with overly reassuring texts) could undermine message credibility and clarity [38].

Hypothesis 3: the impact of emoji valence on preventive behavioral intentions is moderated by emoji-text congruence; specifically, negative emojis will be most effective when paired with text that is congruent in conveying risk or threat, while the persuasive effectiveness of positive emojis may be diminished if this text conveys negative emotional framing.

**Figure 1.**
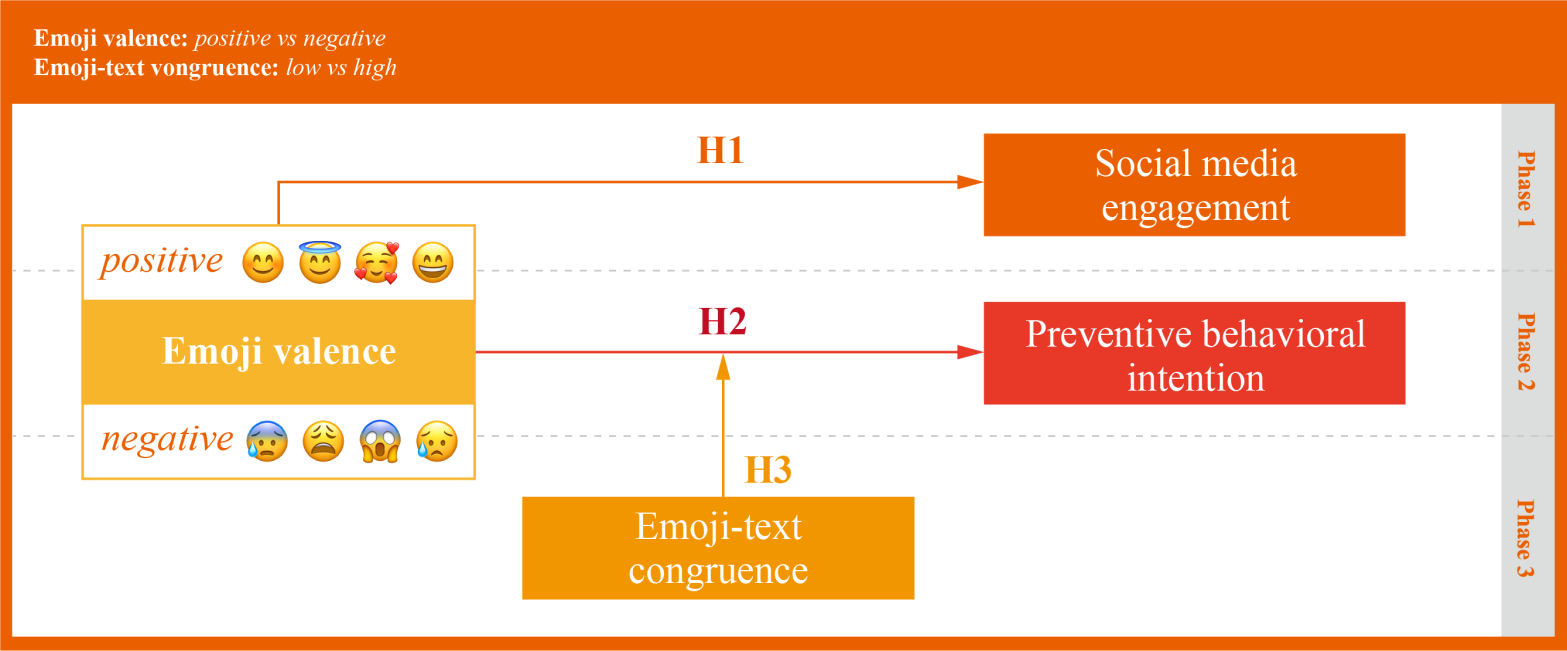
The conceptual model and phase hypotheses.

The conceptual model of this study is presented in Figure 1.

## Methods

### Overview

To systematically test these hypotheses, we used a multimethod approach comprising an automated content analysis of real-world social media data and 2 online simulation experiments. To enhance the ecological validity of the study, we examined or simulated three phases of messaging-based health communication interventions across different social media platforms (Twitter, Facebook, and WeChat). These three phases cover both emergency public health crises and regular physical examination scenarios. This robust approach enables a comprehensive examination of how emoji valence and congruence impact both social media engagement and preventive behavioral intentions in health communication.

### Ethical Considerations

This research received approval from the Academic Ethics Review Committee of the College of Fine Arts, Guangdong Polytechnic Normal University (approval number GPNU-GD25YYS34). Before participation, all participants were presented with an electronic informed consent form on the Credamo [47] platform describing the study purpose, voluntary nature of participation, data use, and compensation. Only those who provided digital consent were allowed to proceed. Participants were informed that they could withdraw from the survey at any time without penalty. All responses were collected anonymously (or de-identified before analysis) and analyzed in aggregate form only, and no personally identifiable information was retained by the research team. Those who completed the survey received compensation of 3 RMB (about US $0.44 as of July 16, 2026). The study complied with applicable institutional and national ethical guidelines for research involving human participants.

### Phase 1

#### Data Collection

To examine how the emotional valence of emojis relates to public engagement with health messages, we collected 320,151 tweets from 48 prominent Twitter (now “X”) accounts specializing in health communication, including the World Health Organization, NPR Health, and NBC News Health (see Multimedia Appendix 1 for the full list). Data were retrieved using the academic research product track of the Twitter application programming interface (API) v2, which provides full-archive access to public tweets. Using the academictwitteR package in R (version 4.3; R Foundation for Statistical Computing), an automated script retrieved all tweets posted by each account between January 1, 2019, and December 31, 2021. This 3-year window was selected to capture health communication dynamics spanning the prepandemic period, the onset of COVID-19, and the ongoing pandemic, thereby enhancing temporal generalizability. For each tweet, the script extracted textual content, posting timestamp, originating account, and engagement metrics (likes, retweets, replies, and quote tweets). During data processing, 2 accounts contributing fewer than 10 tweets over the study period were excluded, yielding a final analytic sample of 46 accounts. Several preprocessing steps were applied to ensure data quality and analytic consistency. First, duplicate tweet records arising from API pagination were removed. Retweets (identified by the “RT @” prefix) were excluded, as their engagement metrics reflect the original author’s content rather than the retweeting account’s communication choices. Tweets in languages other than English were filtered out using the language metadata provided by the Twitter API, and tweets with fewer than 10 characters or empty text fields were discarded. Finally, to mitigate the influence of viral outliers on model estimates, tweets exceeding the 99th percentile on any individual engagement metric (likes, retweets, replies, or quote tweets) were trimmed. After these steps, 257,648 tweets remained for analysis (See Multimedia Appendix 2 for detailed documentation).

#### Sentiment Classification

The emotional valence of emojis within each tweet was classified using a three-stage automated cascade. First, each emoji character was scored using the VADER (Valence Aware Dictionary and Sentiment Reasoner) sentiment analyzer via the *VADER R* package, similar to the Emoji Sentiment Ranking [15]. When VADER could not assign a nonzero score to the raw character, the emoji’s standardized Unicode name was retrieved using the emoji R package and re-scored through VADER. For the remaining unscored emojis, a keyword-matching heuristic compared the emoji name against curated lists of positive and negative semantic patterns. Emojis scoring above +0.05 were labeled positive, below −0.05 negative, and between these thresholds neutral. At the tweet level, tweets were then categorized into five groups based on the composition of their emoji labels: positive (n=16,861), negative (n=1312), neutral (n=13,470), mixed (containing emojis of differing valences; n=528), or none (no emojis present; n=225,477).

#### Measures and Variables

The dependent variable was social media engagement, operationalized as the sum of likes, retweets, replies, and quote tweets [26,27]. This composite score was log-transformed using log (x+1) to address the pronounced right-skew typical of engagement distributions [27]. We additionally analyzed each component—log likes, log retweets, and log replies—separately to assess consistency across engagement types. The independent variable was emoji valence, coded as a five-level categorical factor (negative, positive, neutral, mixed, or none). To strengthen internal validity, five tweet-level covariates were included: tweet character length, URL presence (binary), hashtag count, mention count, and a weekend posting indicator. Variance inflation factors ranged from 1.02 to 1.31, indicating no multicollinearity (Multimedia Appendix 2).

#### Analytical Approach

Because tweets are nested within accounts that vary in audience size, topical focus, and credibility, we used hierarchical linear models (HLM) with random intercepts for accounts. This approach partitions engagement variance into within-account and between-account components, preventing the conflation of emoji effects with account-level characteristics (see Multimedia Appendix 2 for model equations). The intraclass correlation coefficient (ICC) was computed from null (intercept-only) models to quantify between-account variation. We followed a sequential model-building strategy: Model 1 (M1) included a binary emoji presence indicator; Model 2 (M2) replaced this with the five-level valence factor; Model 3 (M3) added covariates to M1; and Model 4 (M4) combined the valence factor with all covariates. M4 served as the primary model for hypothesis testing. Models were estimated via restricted maximum likelihood (REML) using the *lme4* package, with Satterthwaite-approximated degrees of freedom from lmerTest. Model fit was compared using AIC and BIC (Multimedia Appendix 2).

### Phase 2

#### Design and Procedure

This phase aimed to investigate the influence of different emoji valence in health messages on the intention to engage in preventive behaviors. An online experiment was conducted in November 2022, simulating a health message when a booster shot of the COVID-19 vaccine had been made available to the public. While ensuring the consistency of the message text, the health message introduced and persuaded participants about the vaccine booster shot by categorizing the emojis into two emotional valences (positive vs negative). Participants were sent a link to a survey in which they were invited to browse a webpage and sign an informed consent form. They were then exposed to one of two stimuli (ie, a positive emoji or a negative emoji). A piece of health information published by a fictitious health organization was displayed to the participants. Participants were asked to carefully read the information and evaluate their intention to receive the booster shot for COVID-19. Finally, the participants reported their demographic information, such as sex, age, and education.

#### Stimulus Material

The selection of several representative emojis was based on the Emoji Sentiment Ranking developed by Kralj Novak et al [15]. We invited four visual design professionals, including two visual designers and two design department teachers from universities. Four experts selected candidate emojis from the list of Apple-device emojis in the Emojipedia database [48]. The selection was based on three criteria: (1) the official descriptions and meanings displayed in the database; (2) the Emoji Sentiment Ranking developed by Kralj Novak et al [15]; and (3) the quality of their semantic and visual representativeness [49]. Ultimately, experts selected 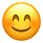 and 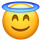 as representatives of positive emojis, and 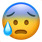 and 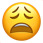 as representatives of negative emojis. After determining the four emoji representatives, we conducted a pretest for manipulation check purposes. The pretest was conducted online using Credamo, and participants were asked to evaluate whether “this message uses positive (or negative) emojis?” using a Likert scale ranging from 1 (strongly disagree) to 7 (strongly agree). We recruited 120 participants for the pretest (mean 29.39, SD 6.58), and 65 (54.2%) of them identified as women. The results of a *t* test showed that, compared to participants who saw negative emojis (mean 2.17, SD 1.77), participants in the positive emoji condition were more likely to believe that the emojis used in the information tended to express positive emotions (mean 6.20, SD 1.15, *t*_118_=−14.8; *P*<.001). In addition, participants who received health information with negative emojis (mean 6.15, SD 1.52) were more likely to believe that the emojis were negative than those in the positive emoji condition (mean 1.67, SD 1.08, *t*_118_=18.6; *P*<.001). These results provide convincing evidence for the manipulation, demonstrating that participants can correctly identify the emotional value of emojis.

We created a fictitious organization named “Health Online” and designed a Facebook post using Adobe Illustrator (Multimedia Appendix 3). The post was displayed in traditional Chinese characters, with the headline and content arranged sequentially from top to bottom. The publishing unit, publishing time, and the body of the information were designed sequentially below. The content presented an overview of COVID-19 vaccine booster shots along with corresponding vaccination recommendations aimed at promoting immunization. Finally, emojis were incorporated into the messages, and the emotional valence of the emojis was manipulated to be either positive or negative. The emojis were positioned within the text, both in the middle and at the end. Apart from the manipulation of emojis, all other content remains consistent.

#### Participants

Phase 2 recruited 220 participants from Hong Kong, Macau, and Taiwan online via Credamo, and assigned them equally to one of the four scenarios. All participants were users of traditional Chinese characters, and 135 (61.4%) of them were women. Participants’ ages ranged from 18 to 45 years old (mean 27.42, SD 6.32). Among them, 180 individuals (81.8%) hold bachelor’s degrees or higher. All participants reported having prior experience reading Facebook posts.

#### Measurement Instrument

The scale to measure vaccination intention was adapted from Gerend and Shepherd’s research [40] and was measured using a 7-point Likert scale. The first four items were anchored from 1 (strongly disagree) to 7 (strongly agree). The last two items were anchored from 1 (very unlikely) to 7 (very likely). These questions examined participants’ willingness to receive the COVID-19 vaccine booster shot in the short and long term ()(Textbox 1). Higher scores indicate stronger preventive behavioral intention. We examined the reliability of the scale in this experiment (*α*=0.919).

Textbox 1. **The reliability of the measurement.**

**Construct:** Preventive Behavioral Intention (Adapted from Gerend & Shepherd [40])

**Items:**

- I will get more information about the COVID-19 vaccine booster shot
- I will consider getting a booster shot
- I will try the booster shot
- I will actually accept the booster shot
- How likely are you to accept the vaccine if hospitals or medical facilities offer the booster shot?
- Will you actively accept the vaccination if the time limit of the booster shot is every 6 months or 8 months?

**Cronbach alpha:** 0.919

### Phase 3

#### Overview

The objective of study 3 was to further demonstrate the interaction effect of emoji valence and emoji-text congruence on health behavioral intentions. Study 3 is a 2 (emoji valence: positive versus negative) by 2 (emoji-text congruence: low versus high) between-subject experiments design.

#### Design and Procedure

Participants were informed that this study was conducted to evaluate information about physical examinations. We used scenarios related to physical examinations for the online experiment in April 2023. After signing the informed consent form, participants were provided with and read a health message about regular blood tests. The emojis embedded in the health message could be either negative or positive. Additionally, the message text was manipulated to have high or low congruence with the negative emojis while ensuring the meaning remained consistent (ie, positive emoji and low congruence, positive emoji and high congruence, negative emoji and low congruence, or negative emoji and high congruence). Participants were first prompted to assess the congruence between the emojis and the text in the health message (ie, “I feel that the emojis in the message are not consistent with the text”). Then their preventive behavioral intention was also assessed (ie, If hospitals or medical institutions offer regular check-up services, how likely am I to undergo regular physical examinations?). At the end of the experiment, participants reported their demographic information.

#### Stimulus Material and Participants

To enhance the generalizability of emojis and the robustness of cross-context in this study, 4 additional representatives, different from those in study 2, were selected for this phase of the experiment. 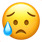 and 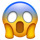 were chosen as positive emojis, while 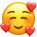 and 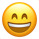 were selected as negative emojis. Selecting additional emojis serves to strengthen external validity. The emotional valence of these emojis was ensured as they were also derived from previous studies [13,16,50]. In addition, to simulate the public’s reception of health messages, we created a fictitious WeChat public account named “Health Smart Butler.” The visualization of its health tweets was designed using Adobe Illustrator (Multimedia Appendix 4). All stimuli were displayed in Chinese, with components such as the title, the message publisher, message release date and time, the purpose of regular physical examination, and the role of blood tests. Next, the congruence of the body content with the negative emojis was categorized into “high” and “low” expressions. The content highlighted the benefits of regular physical examination, providing assurance for one’s life. Moreover, the importance and usefulness of blood tests in these examinations were presented (specific stimulus texts are shown in the table below). To manipulate the emoji-text congruence, we placed the emoji in different locations. For the high fit with negative emojis condition, emojis were placed after negative words such as risks, problems, lacking. In contrast, in the low fit with negative emojis condition, emojis were displayed next to neutral or positive words such as tests, healthy, crucial, testing. Please see Table 1 for detailed information.

A total of 190 online participants from mainland China were recruited through Credamo and randomly assigned to each experimental group. Participants’ ages ranged from 19 to 57 years (mean 30.19, SD 6.62), with 113 (59.5%) identified as women. Among them, 174 individuals (91.6%) had completed a bachelor’s degree or higher. All participants reported having prior experience reading health messages through WeChat public account posts.

**Table 1.** The text of the experimental stimulus.

Congruence	High (low) fit with negative (positive) emojis	Low (high) fit with negative (positive) emojis
Title	Regular blood tests, expose hidden health risks [emoji]	Regular blood tests [emoji], expose hidden health risks
Content	The main purpose of regular health check-ups for healthy people is to timely reveal potential health risks [emoji] and prevent the occurrence of diseases. Ignoring regular specialized check-ups may lead to early signs of diseases being overlooked, bringing unnecessary risks to your healthy life. In the annual health check-up, blood tests are especially crucial. Not having a blood test could mean that you are unaware of your own health condition, missing the key moment to identify potential common health problems [emoji]. During blood collection, multiple tubes are usually used for testing; missing the check-up could mean missing the chance to test your blood count, making it impossible to detect whether your body is lacking in iron, vitamins, etc [emoji] [emoji] [emoji]	The main purpose of regular health check-ups for healthy individuals [emoji] is to timely reveal potential health risks and prevent the occurrence of diseases. Ignoring regular specialized check-ups may lead to early signs of diseases being overlooked, bringing unnecessary risks to your healthy life. In the annual health check-up, blood tests are especially crucial [emoji]. Not having a blood test could mean that you are unaware of your own health condition, missing the key moment to identify potential common health problems. During blood collection, multiple tubes are usually used for testing [emoji] [emoji] [emoji]; missing the check-up could mean missing the chance to test your blood count, making it impossible to detect whether your body is lacking in iron, vitamins, etc

## Results

### Phase 1

The final dataset comprised 257,648 tweets from 46 health-focused Twitter accounts. Of these, 32,171 (12.5%) contained at least one emoji: 16,861 positive (6.5% of all tweets), 13,470 neutral (5.2%), 1312 negative (0.5%), and 528 mixed-valence (0.2%). Overall, tweets received a median of 12 total engagements (mean 32.22, SD 49.96). Descriptively, tweets with negative emojis received higher mean likes (mean 39.5, SD 49.5) than tweets with positive emojis (mean 16.4, SD 36.4) or no emojis (mean 20.6, SD 32.5). Full descriptive statistics are presented in Multimedia Appendix 2.

Null-model ICCs indicated that between-account differences explained 48.5% of the variance in log total engagement, 46.1% in log likes, 45.4% in log retweets, and 28.4% in log replies, confirming the necessity of multilevel modeling.

Table 2 presents the primary hypothesis-testing model (M4), incorporating emoji valence and all covariates, for each engagement outcome. For log total engagement, tweets containing negative emojis received significantly higher engagement than tweets without emojis (*β*=0.25, *SE*=0.02, *t*_257,596.35_=10.44; *P*<.001). Neutral (*β*=0.23, *SE*=0.01; *P*<.001) and mixed (*β*=0.36, *SE*=0.04; *P*<.001) emojis were also positively associated with engagement, whereas positive emojis showed no significant difference from emoji-free tweets (*β*=0.01, *SE*=0.01, *t*_257, 610.93_=1.52; *P*=.13). Critically, a planned contrast confirmed that negative-emoji tweets generated significantly higher engagement than positive-emoji tweets (Δ*β*=0.24, *SE*=0.03, *t*_257,595.11_=9.59; *P*<.001), directly supporting H1. These patterns were consistent across engagement sub-metrics: negative emojis outperformed positive emojis in log likes (Δ*β*=0.17, *t*_257, 595.36_=6.70; *P*<.001), log retweets (Δ*β*=0.29, *t*_257, 595.71_=13.90; *P*<.001), and log replies (Δ*β*=0.14, *t*_257, 599.22_=9.24; *P*<.001). Among covariates, URL presence was the strongest predictor (*β*=0.93; *P*<.001), followed by weekend posting (*β*=0.11; *P*<.001), hashtag count (*β*=0.07; *P*<.001), and tweet length (*β*=0.01; *P*<.001), while mention count was negatively associated with engagement (*β*=−0.09; *P*<.001). The inclusion of covariates in M4 improved model fit substantially over M2 (ΔAIC=−47,903 for log total engagement), yet the negative emoji advantage persisted, indicating that the effect is not attributable to the tweet-level characteristics examined.

**Table 2.** Sequential model results for log total engagement. Reference category for emoji valence is “No Emoji.” M4 is the primary hypothesis-testing model. The notable sign change for the positive-emoji coefficient from M2 (−0.243) to M4 (+0.012, n.s.) indicates that the apparent negative association of positive emojis in M2 was confounded with tweet characteristics (eg, positive-emoji tweets tended to contain fewer URLs and be shorter).

Predictor	M1: emoji presence	M2: emoji valence	M3: presence+ covariates	M4: valence + covariates
Intercept, *β* (SE)	1.93 (0.14)^b^	1.92 (0.14)^b^	0.51 (0.14)^b^	0.53 (0.14)^a^
Has emoji (binary), *β* (SE)	0.07 (0.01)^b^	—	0.12 (0.01)^b^	—
Negative, *β* (SE)	—	0.33 (0.023)^b^	—	0.25 (0.02)^b^
Neutral, *β* (SE)	—	0.40 (0.01)^b^	—	0.23 (0.01)^b^
Mixed, *β* (SE)	—	0.31 (0.04)^b^	—	0.36 (0.04)^b^
Positive, *β* (SE)	—	−0.24 (0.01)^b^	—	0.01 (0.01)
Tweet length, *β* (SE)	—	—	<0.01 (<0.001)^b^	<0.01 (<0.001)^b^
URL present, *β* (SE)	—	—	0.95 (0.01)^b^	0.93 (0.01)^b^
Hashtag count, *β* (SE)	—	—	0.07 (<0.01)^b^	0.07 (<0.01)^b^
Mention count, *β* (SE)	—	—	−0.09 (<0.01)^b^	−0.09 (<0.01)^b^
Weekend, *β* (SE)	—	—	0.11 (<0.01)^b^	0.11 (<0.01)^b^
σ² (account)	0.85	0.84	0.90	0.90
σ² (residual)	0.90	0.89	0.74	0.74
Akaike information criterion	705,115	701,986	654,547	654,083
Bayesian information criterion	705,157	702,059	654,641	654,208

a *P*<.05.

b *P*<.001.

**Figure 2.**
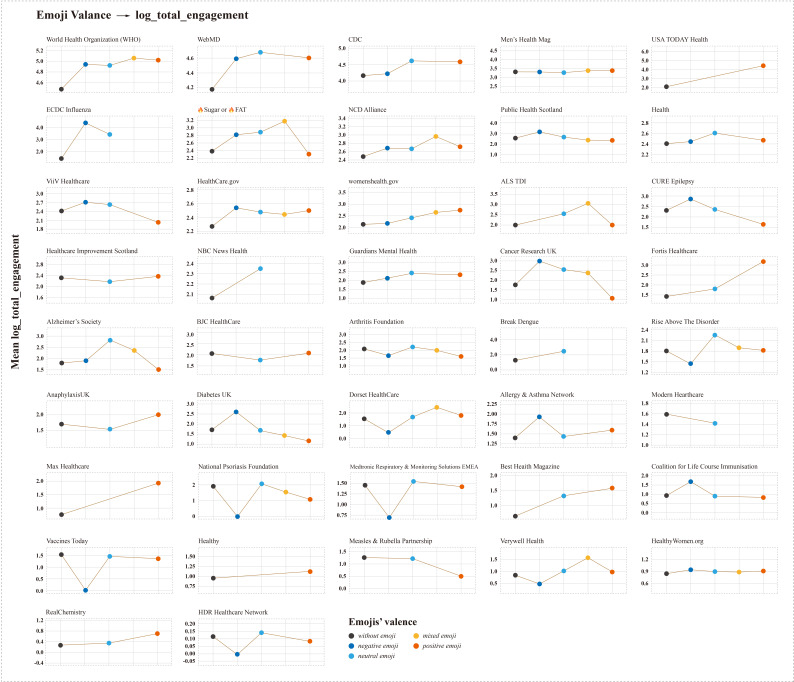
The multilevel effect of emoji valence on engagement. Four accounts were excluded from the figure due to the absence of tweets containing both negative and positive emojis.

Although the pooled multilevel models show a clear average advantage for negative emojis, the account-specific patterns are not perfectly uniform. To address this heterogeneity, we examined per-account estimates of the negative-emoji coefficient (relative to positive or to no-emoji, depending on the model specification). Most accounts showed a positive association consistent with the pooled effect (Figure 2), but a small subset diverged from this pattern. Specifically, the negative-emoji effect was statistically indistinguishable from zero for accounts such as the “Health,” “Arthritis Foundation,” and “CDC,” and it was directionally reversed for accounts like “WHO” and “National Psoriasis Foundation,” where negative-emoji tweets were associated with lower engagement than comparable posts with positive emojis.

### Phase 2

A series of 2-tailed *t* tests were used to analyze the results. The result of the manipulation check showed that, compared to information with negative emojis (mean 2.32, SD 1.76), receivers perceived a post with positive emojis to contain positive emotions (mean 4.78, SD 1.75, *t*_218_= −10.4; *P*<.001). Therefore, the manipulation of emojis in the experiment was successful.

We conducted another independent-sample 2-tailed *t* test to determine the effects of emoji valence on the intention to receive a booster shot. The results (Table 3) indicated a significant main effect of emoji valence on booster shot intention (*t*_218_= −4.15; *P*<.001). Specifically, using negative emojis (mean 5.61, SD 0.99) increased participants’ intention to receive booster shots more than using positive emojis (mean 4.88, SD 1.53). These findings support hypothesis 2 and, simultaneously, supplement the conclusions of study 1. The use of negative emojis in health messages not only enhances social media engagement but also increases people’s willingness to receive booster shots.

**Table 3.** The results of *t* test.

Group	Mean (SD)	*t* test (*df*)
Preventive behavioral intention		218 (−4.15)^a^
Negative emojis (N=110)	5.61 (0.99)	
Positive emojis (N=110)	4.88 (1.53)	

a *P*<.001*.*

### Phase 3

The results of a *t* test revealed that participants who received the messages with positive emojis (mean 6.34, SD 0.82) were more likely to perceive the emoji as conveying more positive emotional valence than did those in the negative emojis condition (mean 1.43, SD 0.72, *t*_186.89_= −43.85; *P*<.001). Compared with participants who received messages containing positive emojis (mean 1.44, SD 0.77), those exposed to messages with negative emojis perceived the emotional valence communicated by the emojis as more negative (mean 6.55, SD 0.67, *t*_186.72_=48.70; *P*<.001). Another manipulation check to assess whether emojis in messages matched the text was appropriately manipulated suggested that in messages with negative emojis, content that was perceived to be low in congruence with negative emojis (mean 4.02, SD 1.73) mismatched negative emojis more than those that were high in congruence with negative emojis (mean 3.11, SD 1.66, *F*_1, 186_=7.62; *P*=.006). Additionally, among messages using positive emojis, content with high congruence to negative emojis (mean 2.82, SD 1.29) was perceived to have a lower match to positive emojis than content with low congruence to negative emojis (mean 3.52, SD 1.64, *F*_1, 186_=4.81; *P*=.03). Therefore, both manipulations were successful.

**Figure 3.**
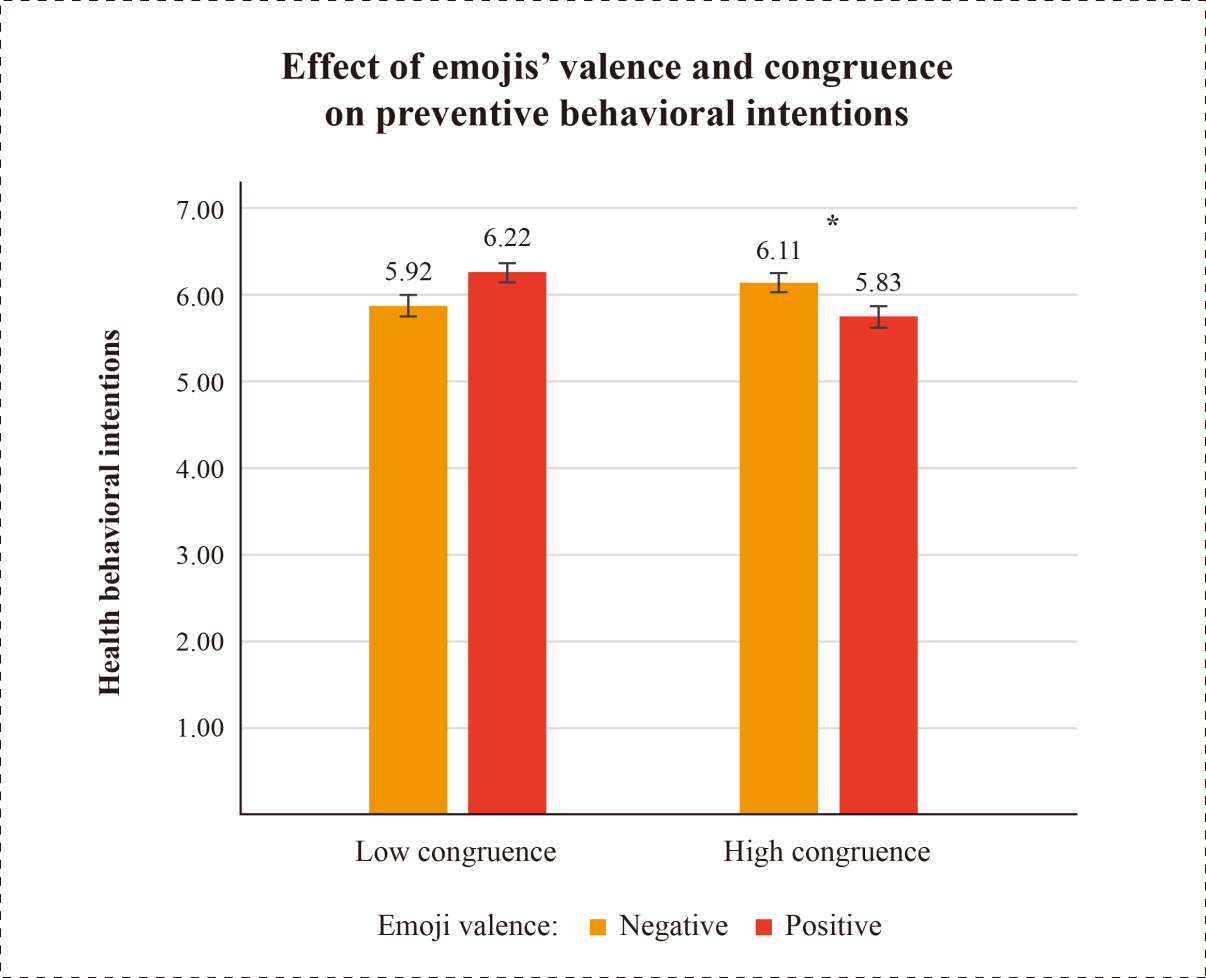
Interaction effect of emojis’ valence and negative emoji-text congruence on preventive behavioral intentions. * *P*<.05.

We conducted a 2-way ANOVA analysis to test hypothesis 3. The results indicated that there was no significant main effect of emojis on preventive behavior intentions (*F*_1, 186_=0.01, *η^2^*<.001; *P*=.91), regardless of whether the health message contained positive (mean 6.03, SD 0.87) or negative emojis (mean 6.01, SD 0.87). On the other hand, the main effect of emoji-text congruence on preventive behavior intentions was also not significant (mean*_low_* 5.97, SD 0.87; mean*_high_* 6.07, SD 0.87, *F*_1, 186_=0.58; *P*=.45). However, interestingly, a significant interaction effect on preventive behavior intentions was observed (*F*_1, 186_=5.46, *η^2^*=.028; *P*<.05), as shown in Table 4 and Figure 3. It showed that in health messages with negative emojis, messages with high negative emoji-text congruence (mean 6.11, SD 0.75) enhance people’s intentions to engage in preventive behaviors more than those with low congruence (mean 5.83, SD 0.95). In contrast, when the emojis in the health messages are positive, participants’ intention to undergo regular physical examinations became less favorable as the negative emoji-text congruence increases (mean*_low_* 6.22, SD 0.74 versus mean*_high_*=5.91, SD 0.97). These findings support hypothesis 3 and, simultaneously, supplement the conclusions of studies 1 and 2. The use of negative emojis in health messages not only enhances social media engagement but also increases people’s preventive behavior intentions, especially when negative emojis have high congruence with the text.

**Table 4.** Descriptive statistics of ANOVA.

Source	*F* (*df*)	*P* value	η^2^
Intention to take regular physical examinations			
Emoji valence	0.01 (1, 186)	.91	<.001
Emoji-text congruence	0.58 (1, 186)	.45	.003
Emoji valence × emoji text congruence	5.46 (1, 186)	.02^a^	.028

a *P*<.05.

## Discussion

### Principal Findings

This study used automated content analysis and experimental methods to examine the role of emojis in health communication. Specifically, we investigated the effects of emojis with different emotional valence on social media engagement, as well as how their congruence with text influences preventive behavioral intentions. Compared to positive emojis, the use of negative emojis in health messages generated more social media engagement and preventive behavioral intentions. Negative emojis demonstrated significantly stronger persuasive effectiveness than positive emojis or the absence of emojis in text-based mHealth interventions during the pandemic. However, this advantage depends on the congruence between the emojis and the accompanying textual message.

### Comparison With Prior Work and Theoretical Contributions

We first indicate that the valence of emojis used in tweets has a significant impact on the social media engagement that tweets receive, even after accounting for the nested data structure through a random-intercept HLM. This finding was also robust when examined using an OLS model with account fixed effects and clustered standard errors. Specifically, tweets that used negative emojis received more social media engagement than tweets that used no emojis or positive emojis. Furthermore, the ICC of approximately 0.49 indicates that a substantial proportion of the variation in social media engagement is attributable to differences between Twitter accounts, underscoring the importance of accounting for this multilevel structure. Consistent with previous studies, unlike daily interactions on social media, negative visuals tend to capture individuals’ attention and interest in health communication [16,35,37,51]. Users tend to interact and share health information with negative emojis, and these visuals with negative emotions evoke rich emotional experiences, including fear [52,53]. This result confirms that the information processing of emojis in health communication on social media provides users with a pathway for emotional reactions. Public health organizations can use emojis in health message design and disseminate them via social media to obtain more comments, likes, and shares, and to increase information coverage.

The findings of further experiments show that the use of negative emojis in health messages can elicit higher preventive behavioral intentions compared to positive emojis. This extends the view of previous research that the valence of emojis can guide individuals’ perceptions and behavioral intentions [16,19,39]. Compared with positive emojis, negative emojis may be more effective in motivating the public to engage in low-risk actions because they have the potential to activate heightened perceptions of risk and fear [14,16,17]. Also, this finding further demonstrates the effectiveness of using emojis for emotional contagion and health persuasion on social media [35,51]. We do not believe that the use of negative emojis in health messages is intended to immerse recipients in negative emotions. Instead, the negative emotions triggered by these emojis should be transformed by individuals into feelings of hope [52,53], thereby emphasizing the importance and necessity of preventive behaviors. This result further complements the emotional pathways provided by emojis in health communication and addresses the need, as proposed by Erle et al [6], to test the applicability of the EASI model within digital communication contexts.

In a postpandemic, regular physical examinations context, emoji valence alone is not sufficient to shape preventive behavioral intentions. The effect of emojis depends on their congruence with the textual framing. The use of negative emojis in health messages can increase preventive behavioral intentions, as demonstrated in the framework of high negative emoji-text congruence. According to the EASI model, the inferential process serves as the primary determinant of the subsequent behavioral effects of emotional expressions when the meanings of emojis are relatively vague and unclear [6]. Our findings also qualify previous work suggesting that a moderate level of incongruity in the schema can enhance evaluations by increasing novelty [45,46]. In the context of preventive health communication, the valence of negative emojis with low congruence to text presentation may appear less likely to be experienced as “interesting” and more likely to be interpreted as confusing or misaligned, thereby weakening individuals’ preventive intentions. Negative emojis in health messages with high emoji-text congruence maximize the consistency among the visual, emotional, and content elements conveyed to the user [16,19,54]. Naturally, individuals are more likely to positively and smoothly accept the emotions elicited by an object with high internal consistency, thereby translating those emotions into preventive behavior intentions. However, when negative emojis are replaced with positive ones, the effects of emotional inference become counterproductive due to the inconsistency in the schema. Negative emojis in a textual context of high negative emoji-text congruence may prompt the audience to devote more cognitive effort in processing the information, thereby increasing preventive behavior intentions. In real-world health communication, it is common for message design components to yield only small-to-moderate effect sizes because outcomes are shaped by multiple influences. However, when health messages are repeatedly delivered to large populations, even modest effects can accumulate and become highly consequential at the population level. Emojis, similar to facial expressions, provide two pathways—emotional reactions and inferential processes—in the dissemination of health information on social media platforms. More specifically, we build on the “emojis as social information” model proposed by Erle et al [6] and extend its applicability to the context of messaging-based interventions in mHealth. Emojis, as multimodal cues that convey both social information and visual semantics, interact with textual health messages to form multimodal content. This integration facilitates the interpretation, interaction, and acceptance of information by individuals on social media platforms.

### Practical Implications

In addition to theoretical refinement, this study also offers several practical recommendations. First, health organizations and online health accounts are encouraged to use emojis as an interactive tool for health communication on social media. Second, using negative emojis not only increases audience engagement but also enhances individuals’ intentions to engage in preventive and health behaviors. Beyond the communication and interaction of health knowledge in daily life, the use of negative emojis is particularly effective for health messages that require strong public attention. For instance, during urgent health crises such as monkeypox or COVID-19 outbreaks, negative emojis can encourage individuals to adopt preventive behaviors like vaccination or promote the spontaneous communication of health messages. Finally, when using negative emojis in health messages, it is crucial to ensure congruence with the textual content. Properly placing emojis can maximize the persuasive power of health messages in mHealth and social media.

### Limitations and Further Research

This study was deliberately designed to span multiple time points, demonstrating that emojis have influence on health communication across different phases of the pandemic and beyond, especially negative emojis. The first 2 phases were conducted in a high‐threat context (COVID-19), but the findings should not be interpreted as evidence that negative emojis are more persuasive than positive emojis across diverse health contexts. By contrast, in postpandemic settings such as regular physical examination scenarios, the persuasive impact of emoji valence depends on its congruence with the accompanying text. These results reveal the boundary conditions for generalizing beyond the pandemic period and highlight the need to explore the role of emojis across a broader range of health risks and cultural contexts. Further research should consider additional specific health contexts, such as sexual and reproductive health, depression, and anxiety. Currently, the study only compares the effects of positive and negative emojis, without examining neutral emojis and combinations of mixed valences. On social media, users often use emojis with different emotional valences to supplement their emotions and textual meanings [25,55,56]. Combinations of emojis with different emotional valences may become a direction for further research. This study relies on social media engagement and preventive behavioral intention as observed outcome indicators. The limitation highlights the need for future research to further test the applicability of the EASI model by incorporating mediating variables (eg, arousal and perceived ambiguity) and simultaneously represents an issue that the field of emoji research seeks to address. In addition, the visual stimuli used in this study were selected from the “Smileys” series of emojis. Future work should consider nonfacial expression emojis, such as gestures, occupations, and actions, to expand the applicability of emoji categories in health communication. Moreover, the study participants focused on the Chinese-speaking region; future studies may investigate the effect of emoji valence in different cultures. Furthermore, the effect of emoji valence may also depend on different languages and texts, as well as the complexity of the information. Future studies may investigate how these contextual factors influence the effect of emoji valence on the public’s perception, understanding, and attitudes.

### Conclusions

This study demonstrates that emojis are not merely decorative elements in digital health communication, but persuasive multimodal cues that can shape both audience engagement and preventive behavioral intentions. Across the pandemic and post-pandemic contexts, negative emojis were found to be especially effective in attracting social media engagement and motivating preventive responses. However, their persuasive advantage is not universal; in lower-threat contexts, such as regular physical examinations, their effectiveness depends on congruence with the accompanying textual framing. These findings extend the applicability of the EASI model to mHealth and social media by showing that emojis influence health communication through both emotional reactions and inferential processing. Overall, this study highlights the strategic value of emoji use in health message design and underscores the importance of aligning visual and textual cues to maximize persuasive impact.

## Supplementary material

10.2196/78824 Multimedia Appendix 1 A list of Twitter (X) accounts.

10.2196/78824 Multimedia Appendix 2 Phase 1.

10.2196/78824 Multimedia Appendix 3 Stimulus (Phase 2).

10.2196/78824 Multimedia Appendix 4 Stimulus (Phase 3).

## Abbreviations

EASI: emotions as social information
ICC: intraclass correlation coefficient
mHealth: mobile health
REML: restricted maximum likelihood
VADER: Valence Aware Dictionary and Sentiment Reasoner

## References

[1] Kaye SA, White MJ, Lewis I (2017). The use of neurocognitive methods in assessing health communication messages: a systematic review. J Health Psychol.

[2] Li YT, Chen ML, Lee HW (2024). Health communication on social media at the early stage of the pandemic: examining health professionals’ COVID-19 related tweets. Soc Sci Med.

[3] King AJ (2015). A content analysis of visual cancer information: prevalence and use of photographs and illustrations in printed health materials. Health Commun.

[4] Heldman AB, Schindelar J, Weaver JB (2013). Social media engagement and public health communication: implications for public health organizations being truly “Social”. Public Health Rev.

[5] Zang S, Zhang X, Xing Y, Chen J, Lin L, Hou Z (2023). Applications of social media and digital technologies in COVID-19 vaccination: scoping review. J Med Internet Res.

[6] Erle TM, Schmid K, Goslar SH, Martin JD (2022). Emojis as social information in digital communication. Emotion.

[7] Ko E (Emily, Kim D, Kim G (2022). Influence of emojis on user engagement in brand-related user generated content. Comput Human Behav.

[8] Murray E, Burns J, See TS, Lai R, Nazareth I (2005). Interactive health communication applications for people with chronic disease. Cochrane Database Syst Rev.

[9] Boender TS, Louis-Ferdinand N, Duschek G (2022). Digital visual communication for public health: design proposal for a vaccinated emoji. J Med Internet Res.

[10] Langdon KJ, Scherzer C, Ramsey S, Carey K, Rich J, Ranney ML (2021). Feasibility and acceptability of a digital health intervention to promote engagement in and adherence to medication for opioid use disorder. J Subst Abuse Treat.

[11] Lotfinejad N, Assadi R, Aelami MH, Pittet D (2020). Emojis in public health and how they might be used for hand hygiene and infection prevention and control. Antimicrob Resist Infect Control.

[12] Sick J, Monteleone E, Pierguidi L, Ares G, Spinelli S (2020). The meaning of emoji to describe food experiences in pre-adolescents. Foods.

[13] Yu S, Zhao L (2022). Designing emotions for health care chatbots: text-based or icon-based approach. J Med Internet Res.

[14] Helme DW, Donohew RL, Baier M, Zittleman L (2007). A classroom-administered simulation of a television campaign on adolescent smoking: testing an activation model of information exposure. J Health Commun.

[15] Kralj Novak P, Smailović J, Sluban B, Mozetič I, Perc M (2015). Sentiment of emojis. PLoS ONE.

[16] Lin TS, Luo Y (2023). Health persuasion through emoji: how emoji interacted with information source to predict health behaviors in COVID-19 situation. SSM Popul Health.

[17] Ray EC, Merle PF (2021). Disgusting face, disease-ridden place?: Emoji influence on the interpretation of restaurant inspection reports. Health Commun.

[18] Benito-Ostolaza JM, Echavarri R, Garcia-Prado A, Oses-Eraso N (2021). Using visual stimuli to promote healthy snack choices among children. Soc Sci Med.

[19] Lin TS, Luo Y (2023). Emoji and visual complexity in health information design: a moderated serial mediation model. Telematics and Informatics.

[20] Pfeifer VA, Armstrong EL, Lai VT (2022). Do all facial emojis communicate emotion? The impact of facial emojis on perceived sender emotion and text processing. Comput Human Behav.

[21] Van Kleef GA (2009). How emotions regulate social life: the emotions as social information (EASI) model. Curr Dir Psychol Sci.

[22] Harrington NG (2015). Introduction to the special issue: message design in health communication research. Health Commun.

[23] Vann RJ, Tanner EC, Kizilova E (2022). Perceived access, fear, and preventative behavior: key relationships for positive outcomes during the COVID-19 health crisis. J Consum Aff.

[24] Yıldırım M, Geçer E, Akgül Ö (2021). The impacts of vulnerability, perceived risk, and fear on preventive behaviours against COVID-19. Psychol Health Med.

[25] Huesch M, Chetlen A, Segel J, Schetter S (2017). Frequencies of private mentions and sharing of mammography and breast cancer terms on Facebook: a pilot study. J Med Internet Res.

[26] Yost E, Zhang T, Qi R (2021). The power of engagement: understanding active social media engagement and the impact on sales in the hospitality industry. Journal of Hospitality and Tourism Management.

[27] Yu S, Hu Y (2020). When luxury brands meet China: the effect of localized celebrity endorsements in social media marketing. Journal of Retailing and Consumer Services.

[28] Baum M, Schäfer M, Kabst R (2016). Modeling the impact of advertisement-image congruity on applicant attraction. Hum Resour Manage.

[29] Mandler G (2014). Affect and Cognition.

[30] Hatfield E, Cacioppo JT, Rapson RL (1993). Emotional contagion. Curr Dir Psychol Sci.

[31] Anderson RC, Pearson PD (1984). Handbook of Reading Research.

[32] OSGOOD CE, TANNENBAUM PH (1955). The principle of congruity in the prediction of attitude change. Psychol Rev.

[33] Baumeister RF, Bratslavsky E, Finkenauer C, Vohs KD (2001). Bad is stronger than good. Review of General Psychology.

[34] Kramer ADI, Guillory JE, Hancock JT (2014). Experimental evidence of massive-scale emotional contagion through social networks. Proc Natl Acad Sci U S A.

[35] Clarke N, Pechey E, Kosīte D (2021). Impact of health warning labels on selection and consumption of food and alcohol products: systematic review with meta-analysis. Health Psychol Rev.

[36] Fan R, Xu K, Zhao J (2018). An agent-based model for emotion contagion and competition in online social media. Physica A: Statistical Mechanics and its Applications.

[37] Brewer NT, Parada H, Hall MG, Boynton MH, Noar SM, Ribisl KM (2019). Understanding why pictorial cigarette pack warnings increase quit attempts. Ann Behav Med.

[38] Hou JR, Kankham S (2022). More than feelings? How Facebook reaction icons affect online users’ behavioral intentions toward online health rumor posts. INTR.

[39] Jaeger SR, Lee SM, Kim KO, Chheang SL, Roigard CM, Ares G (2018). CATA and RATA questions for product-focused emotion research: five case studies using emoji questionnaires. Food Qual Prefer.

[40] Gerend MA, Shepherd JE (2012). Predicting human papillomavirus vaccine uptake in young adult women: comparing the health belief model and theory of planned behavior. Ann Behav Med.

[41] Guidry JPD, Carlyle KE, LaRose JG, Perrin P, Messner M, Ryan M (2019). Using the health belief model to analyze Instagram posts about zika for public health communications. Emerg Infect Dis.

[42] King AJ, Lazard AJ (2020). Advancing visual health communication research to improve infodemic response. Health Commun.

[43] Slovic P, Peters E, Finucane ML, Macgregor DG (2005). Affect, risk, and decision making. Health Psychol.

[44] Peracchio LA, Tybout AM (1996). The moderating role of prior knowledge in schema-based product evaluation. J CONSUM RES.

[45] Meyers-Levy J, Tybout AM (1989). Schema congruity as a basis for product evaluation. J CONSUM RES.

[46] Cheong Y, Kim K (2011). The interplay between advertising claims and product categories in food advertising: a schema congruity perspective. Journal of Applied Communication Research.

[47] Credamo.

[48] Emojipedia.

[49] Stoianov D, Kemp N, Wegener S, Beyersmann E (2025). Emojis and affective priming in visual word recognition. Cogn Emot.

[50] Spinelli S, Masi C, Zoboli GP, Prescott J, Monteleone E (2015). Emotional responses to branded and unbranded foods. Food Qual Prefer.

[51] Stonbraker S, Porras T, Schnall R (2020). Patient preferences for visualization of longitudinal patient-reported outcomes data. J Am Med Inform Assoc.

[52] Nabi RL, Myrick JG (2019). Uplifting fear appeals: considering the role of hope in fear-based persuasive messages. Health Commun.

[53] Nabi RL (2015). Emotional flow in persuasive health messages. Health Commun.

[54] Herrando C, Constantinides E (2021). Emotional contagion: a brief overview and future directions. Front Psychol.

[55] Ricard BJ, Marsch LA, Crosier B, Hassanpour S (2018). Exploring the utility of community-generated social media content for detecting depression: an analytical study on Instagram. J Med Internet Res.

[56] Szeto MD, Barber C, Ranpariya VK (2022). Emojis and emoticons in health care and dermatology communication: narrative review. JMIR Dermatol.

