# Supplementary material for: Strategic Use of Negative Emojis in Messaging-Based Interventions for Public Health Communication on Social Media: Mixed Methods Study
Source: JMIR Hum Factors. 2026 Jul 31;13:e78824. doi: 10.2196/78824 (PMC13427074; doi:10.2196/78824)
Supplement: Multimedia Appendix 1 [file humanfactors-v13-e78824-s001.docx]

**Multimedia Appendix 1.** A list of Twitter (X) accounts.

| **Name** | **Account** | **Number of followers** | **ID** | **Name** | **Account** | **Number of followers** | **ID** | **Name** | **Account** | **Number of followers** | **ID** |
| --- | --- | --- | --- | --- | --- | --- | --- | --- | --- | --- | --- |
| **World Health Organization (WHO)** | WHO | 12.1M | 14499829 | **NBC News Health** | NBCNewsHealth | 991.8K | 11856422 | **NPR Health News** | NPRHealth | 1.02M | 13787352 |
| **HealthCare.gov** | HealthCareGov | 284.9K | 86697288 | **Health** | health_com_ | 3.5M | 15566901 | **Best Health Magazine** | besthealthmag | 181.2K | 14869478 |
| **RealChemistry** | MyRealChemistry | 2,070 | 611693226 | **BBC Health News** | bbchealth | 2.3M | 621543 | **USA TODAY Health** | USATODAYhealth | 337.8K | 26077026 |
| **womenshealth.gov** | womenshealth | 882.7K | 6450322 | **National Psoriasis Foundation** | NPF | 23,8K | 17053094 | **Men’s Health Mag** | MensHealthMag | 4.5M | 25093616 |
| **TIME Health** | TIMEHealth | 712.8K | 15723971 | **CDC** | CDCgov | 5.4M | 146569971 | **Modern Healthcare** | modrnhealthcr | 149.8K | 18935711 |
| **Max Healthcare** | MaxHealthcare | 19.4K | 39731064 | **BJC HealthCare** | BJC_HealthCare | 11.9K | 24211863 | **Fortis Healthcare** | fortis_hospital | 101.3K | 39211877 |
| **Dorset HealthCare** | DorsetHealth | 8,739 | 23780823 | **Healthcare Improvement Scotland** | online_his | 22K | 519343797 | **Public Health Scotland** | P_H_S_Official | 48.9K | 742543573 |
| **ViiV Healthcare** | ViiVHC | 20.2K | 3019793950 | **HDR Healthcare Network** | HDRHealthcare | 51 | 1098689849689817089 | **WebMD** | WebMD | 3.1M | 25928253 |
| **Guardians Mental Health** | GuardiansMH | 8248 | 900369765885202433 | **Rise Above The Disorder** | YouAreRAD | 32.5K | 883344936 | **Verywell Health** | Verywell | 18.6K | 15273508 |
| **🔥Sugar or 🔥FAT** | drandyphung | 48.5K | 1082433813534437382 | **Healthy** | healthy | 28.9K | 67113127 | **HealthyWomen.org** | HealthyWomen | 19.6K | 18483313 |
| **Coalition for Life Course Immunisation** | C_L_C_I | 144 | 1455098341331701764 | **VaccinesToday** | VaccinesToday | 20.9K | 205670342 | **Cancer Research UK** | CR_UK | 341K | 20693661 |
| **ALS TDI** | ALSTDI | 9,980 | 191905736 | **Alzheimer's Society** | alzheimerssoc | 201.9K | 34267043 | **Allergy & Asthma Network** | AllergyAsthmaHQ | 9,805 | 23939188 |
| **AnaphylaxisUK** | AnaphylaxisUK | 10.1K | 28312346 | **Arthritis Foundation** | ArthritisFdn | 41K | 17076146 | **CURE Epilepsy** | CureEpilepsy | 19.6K | 259959379 |
| **Breast Cancer** | breastcancer | 21.7K | 26895297 | **Break Dengue** | BreakDengue | 4,322 | 1513673754 | **ECDC Influenza** | ECDC_Flu | 7,943 | 1371668696 |
| **Medtronic Respiratory & Monitoring Solutions EMEA** | MDT_RMS_EMEA | 565 | 1081190828939362305 | **~~Ebola Deeply~~** | Eboladeeply | 6,374 | 2775968243 | **NCD Alliance** | ncdalliance | 33.3K | 242496394 |
| **Diabetes UK** | DiabetesUK | 186.6K | 16864209 | **Measles & Rubella Partnership** | MeaslesRubella | 3,145 | 351342476 | **~~Los factores de la amebiasis~~** | LAmebiasis | 5 | 1459216290942488576 |

*Note.* 2 accounts were removed after the data cleaning process (marked in gray).
