# Supplementary material for: Strategic Use of Negative Emojis in Messaging-Based Interventions for Public Health Communication on Social Media: Mixed Methods Study
Source: JMIR Hum Factors. 2026 Jul 31;13:e78824. doi: 10.2196/78824 (PMC13427074; doi:10.2196/78824)
Supplement: Multimedia Appendix 2 [file humanfactors-v13-e78824-s002.docx]

**Multimedia Appendix 2. Phase 1 Supplementary Materials**

**1. data cleaning process**

We apply six sequential filters, each one operating on whatever survives the previous filter. Here's the cascade:

**Filter 1 — Deduplicate by tweet_id.** If the Academic Twitter API returned any duplicate tweet objects (which can happen when paginating or when a tweet matches multiple query windows), they're collapsed to one row. For a well-behaved scrape this usually removes very little, but if you had overlapping date windows or restarted the scrape, it could be meaningful.

**Filter 2 — Remove retweets** (Config$REMOVE_RETWEETS = TRUE). This drops every row where the text starts with "RT @". For health organization accounts, this is likely your single largest source of data loss. Institutional accounts like WHO, CDCgov, and BJC_HealthCare frequently retweet partner organizations, press releases, and regional health authorities. It's not unusual for 30–50% of an institutional account's timeline to be retweets. The rationale for removing them is sound — retweets reflect the original author's engagement, not the retweeting account's content choices — but it can easily cut your dataset in half.

**Filter 3 — Language filter** (lang == "en"). This keeps only English-language tweets. Accounts like WHO tweet in multiple languages, and some of your other accounts (MaxHealthcare, fortis_hospital) may post in Hindi or other languages. If the lang column is missing or NA for some tweets, those rows are also dropped because NA == "en" evaluates to NA, which filter() treats as FALSE. This is a subtle but potentially significant source of loss — you should check how many tweets have lang == NA in your 01_loaded checkpoint.

**Filter 4 — Minimum tweet length** (tweet_length >= 10). This removes tweets shorter than 10 characters. After retweet removal, you might have some stub tweets (e.g., just a URL, just a mention, or just an emoji) that fall below this threshold. The loss here is usually small but nonzero.

**Filter 5 — Non-empty text.** After str_squish(), any tweet that collapses to an empty string is dropped. This catches edge cases the length filter might miss (e.g., tweets that were only whitespace or special characters).

**Filter 6 — Outlier trimming on engagement metrics.** This is the second-largest source of data loss after retweet removal, and it's more aggressive than it might appear at first glance. The code loops over five columns sequentially: like_count, retweet_count, reply_count, quote_count, and total_engagement. For each column, it computes the 99th percentile of the *current* dataset and removes every row exceeding that threshold. Because the filters are applied in sequence rather than simultaneously, the losses compound. A tweet that's in the top 1% for likes *and* the top 1% for retweets gets caught by the first filter, but then the thresholds are recalculated on the reduced dataset for the next metric, potentially catching tweets that were previously below the 99th percentile. In the worst case, you could lose noticeably more than 5% of your data across the five passes. Viral tweets from accounts like WHO or CDCgov (which can get tens of thousands of likes on a single pandemic-related tweet) are particularly likely to be trimmed here.

**2. HLM Specification**

The two-level structure is specified as follows. At Level 1 (tweet *i* nested within account *j*): $\text{LogEngagement}_{ij}=\beta_{0j}+\beta_{1}\text{Negative}_{ij}+\beta_{2}\text{Neutral}_{ij}+\beta_{3}\text{Mixed}_{ij}+\beta_{4}\text{Positive}_{ij}+\boldsymbol{\gamma}^{'}\mathbf{X}_{ij}+r_{ij}$

where $\mathbf{X}_{ij}$is the vector of tweet-level covariates and $r_{ij}\sim N(0,\sigma^{2})$.

At Level 2 (account *j*):

$$\beta_{0j}=\gamma_{00}+u_{0j},u_{0j}\sim N(0,\tau_{00}^{2})$$

The ICC is computed as $\rho=\tau_{00}^{2}/(\tau_{00}^{2}+\sigma^{2})$.

**3. Hierarchical Linear Models Predicting Social Media Engagement by Emoji Valence**

**Table 1.** Hierarchical Linear Models Predicting Social Media Engagement by Emoji Valence.

| Predictor (Ref: No Emoji) | Log Likes | Log Retweets | Log Replies | Log Total Engagement |
| --- | --- | --- | --- | --- |
| Intercept | 0.300 (0.133)* | −0.010 (0.105) | 0.186 (0.049)*** | 0.534 (0.140)* |
| Emoji valence |  |  |  |  |
| Negative | 0.244 (0.024)*** | 0.223 (0.020)*** | 0.125 (0.015)*** | 0.252 (0.024)*** |
| Neutral | 0.239 (0.008)*** | 0.163 (0.007)*** | 0.098 (0.005)*** | 0.232 (0.008)*** |
| Mixed | 0.363 (0.038)*** | 0.225 (0.032)*** | 0.145 (0.023)*** | 0.358 (0.038)*** |
| Positive | 0.077 (0.008)*** | −0.069 (0.007)*** | −0.015 (0.005)** | 0.012 (0.008) |
| Covariates |  |  |  |  |
| Tweet length | 0.003 (<0.001)*** | 0.002 (<0.001)*** | 0.001 (<0.001)*** | 0.003 (<0.001)*** |
| URL present | 0.850 (0.007)*** | 0.759 (0.006)*** | −0.025 (0.004)*** | 0.934 (0.007)*** |
| Hashtag count | 0.070 (0.002)*** | 0.051 (0.002)*** | −0.003 (0.001)† | 0.068 (0.002)*** |
| Mention count | −0.082 (0.002)*** | −0.070 (0.001)*** | −0.029 (0.001)*** | −0.088 (0.002)*** |
| Weekend | 0.125 (0.004)*** | 0.078 (0.004)*** | 0.022 (0.003)*** | 0.107 (0.004)*** |
| Negative − Positive contrast | 0.166 (0.025)*** | 0.292 (0.021)*** | 0.139 (0.015)*** | 0.240 (0.025)*** |
| Random effects |  |  |  |  |
| σ² (account) | 0.809 | 0.506 | 0.110 | 0.901 |
| σ² (residual) | 0.731 | 0.523 | 0.269 | 0.740 |
| ICC (null model) | .461 | .454 | .284 | .485 |
| AIC | 650,925 | 564,562 | 393,254 | 654,083 |
| Observations | 257,648 | 257,648 | 257,648 | 257,648 |
| Accounts | 46 | 46 | 46 | 46 |

*Notes.* Unstandardized coefficients with standard errors in parentheses. "No Emoji" is the reference category for emoji valence. The Negative − Positive contrast was computed from the model coefficients and their covariance. ICC values are from null (intercept-only) models. †*p* < .10, **p* < .05, ***p* < .01, ****p* < .001.

**4. Model Fit Comparison Across All Outcomes**

**Table 2.** AIC and BIC for All Models and Outcomes.

| **Outcome** | **M1** | **M2** | **M3** | **M4** |
| --- | --- | --- | --- | --- |
| **AIC** |  |  |  |  |
| Log likes | 693,840 | 691,515 | 651,173 | 650,925 |
| Log retweets | 610,744 | 607,190 | 565,306 | 564,562 |
| Log replies | 395,880 | 395,318 | 393,585 | 393,254 |
| Log total engagement | 705,115 | 701,986 | 654,547 | 654,083 |
| **BIC** |  |  |  |  |
| Log likes | 693,882 | 691,589 | 651,267 | 651,050 |
| Log retweets | 610,786 | 607,263 | 565,400 | 564,688 |
| Log replies | 395,922 | 395,391 | 393,679 | 393,379 |
| Log total engagement | 705,157 | 702,059 | 654,641 | 654,208 |

*Note.* M4 provides the best fit for every outcome. The largest improvement occurs from M2 to M3/M4 (addition of covariates).

**5. Pairwise Comparisons (Tukey HSD) for Log Total Engagement**

**Table 3.** Tukey HSD Post-Hoc Comparisons for Log Total Engagement (Unadjusted Model).

| **Comparison** | **Difference** | **95% CI** | ***p*** |
| --- | --- | --- | --- |
| Negative − None | 0.552 | [0.445, 0.659] | < .001 |
| Neutral − None | 0.830 | [0.795, 0.864] | < .001 |
| Mixed − None | −0.053 | [−0.221, 0.116] | .914 |
| Positive − None | −0.814 | [−0.845, −0.783] | < .001 |
| Neutral − Negative | 0.278 | [0.166, 0.389] | < .001 |
| Mixed − Negative | −0.605 | [−0.804, −0.405] | < .001 |
| Positive − Negative | −1.366 | [−1.477, −1.255] | < .001 |
| Mixed − Neutral | −0.882 | [−1.054, −0.711] | < .001 |
| Positive − Neutral | −1.644 | [−1.688, −1.599] | < .001 |
| Positive − Mixed | −0.761 | [−0.932, −0.591] | < .001 |

*Note.* These comparisons are from a one-way model without covariates. The key contrast (Positive − Negative = −1.366, *p* < .001) confirms H1 directionally; with covariates in M4, the difference is attenuated but remains significant (Δβ = 0.240, *p* < .001).

**6. OLS Robustness Check**

**Table 4.** OLS Regression with Account Fixed Effects and Clustered Standard Errors.

| **Predictor** | **Log Likes *β* (SE)** | **Log Retweets *β* (SE)** | **Log Replies *β* (SE)** | **Log Total Engagement *β* (SE)** |
| --- | --- | --- | --- | --- |
| Has emoji | 0.436 (0.205)* | 0.149 (0.159) | 0.138 (0.124) | 0.338 (0.188)† |
| Tweet length | 0.002 (0.002) | 0.003 (0.002)† | 0.001 (0.001) | 0.003 (0.002) |
| URL present | 1.188 (0.386)** | 1.024 (0.261)*** | 0.025 (0.095) | 1.334 (0.390)*** |
| Hashtag count | −0.130 (0.136) | −0.068 (0.111) | −0.026 (0.044) | −0.125 (0.135) |
| Mention count | −0.012 (0.077) | −0.037 (0.067) | 0.008 (0.025) | −0.027 (0.078) |
| Weekend | 0.259 (0.102)* | 0.109 (0.086) | 0.017 (0.030) | 0.204 (0.101)* |

*Note.* M4 provides the best fit for every outcome. The largest improvement occurs from M2 to M3/M4 (addition of covariates).

**7. Negative Binomial Robustness Check**

**Table 5.** Negative Binomial Regression on Raw Engagement Counts.

| **Predictor** | **Likes *β* (SE)** | **Retweets *β* (SE)** | **Replies *β* (SE)** | **Total Engagement *β* (SE)** |
| --- | --- | --- | --- | --- |
| Has emoji | 0.327 (0.009)*** | 0.078 (0.009)*** | 0.368 (0.011)*** | 0.253 (0.008)*** |
| Tweet length | 0.004 (<0.001)*** | 0.006 (<0.001)*** | 0.003 (<0.001)*** | 0.004 (<0.001)*** |
| URL present | 0.640 (0.009)*** | 0.992 (0.009)*** | 0.002 (0.012) | 0.704 (0.008)*** |
| Hashtag count | −0.072 (0.003)*** | <0.001 (0.003) | 0.016 (0.004)*** | −0.049 (0.003)*** |
| Mention count | −0.055 (0.003)*** | −0.056 (0.003)*** | −0.011 (0.004)** | −0.056 (0.003)*** |
| Weekend | 0.230 (0.007)*** | 0.129 (0.007)*** | 0.024 (0.010)* | 0.193 (0.007)*** |

*Notes.* Coefficients are on the log-count scale. All models confirm the significant positive association between emoji presence and engagement, consistent with the HLM findings. **p* < .05, ***p* < .01, ****p* < .001.

**8. Variance Inflation Factors**

**Table 6.** VIF for Covariates in Model 4.

| **Variable** | **VIF** |
| --- | --- |
| Emoji presence (or valence) | 1.10 |
| Tweet length | 1.31 |
| URL present | 1.25 |
| Hashtag count | 1.24 |
| Mention count | 1.25 |
| Weekend | 1.02 |

*Note.* All VIFs are well below the conventional threshold of 5, indicating no multicollinearity concern.

**9. Correlation Matrix**

**Table 8.** Bivariate Correlations Among Key Variables.

|  | **1** | **2** | **3** | **4** | **5** | **6** | **7** | **8** |
| --- | --- | --- | --- | --- | --- | --- | --- | --- |
| 1. Has emoji | — |  |  |  |  |  |  |  |
| 2. Emoji count | .750 | — |  |  |  |  |  |  |
| 3. Log likes | .019 | .078 | — |  |  |  |  |  |
| 4. Log retweets | −.040 | .032 | .863 | — |  |  |  |  |
| 5. Log replies | .074 | .114 | .535 | .502 | — |  |  |  |
| 6. Log total engagement | −.013 | .057 | .979 | .920 | .565 | — |  |  |
| 7. Tweet length | .061 | .109 | .103 | .191 | .121 | .127 | — |  |
| 8. Hashtag count | .103 | .115 | −.020 | .043 | .022 | −.006 | .426 | — |
| 9. Mention count | .183 | .131 | −.084 | −.105 | .047 | −.106 | .223 | .110 |

*Note.* N = 257,648. All correlations ≥ |.006| are significant at *p* < .001 given the large sample size.
