# Supplementary material for: Strategic Use of Negative Emojis in Messaging-Based Interventions for Public Health Communication on Social Media: Mixed Methods Study
Source: JMIR Hum Factors. 2026 Jul 31;13:e78824. doi: 10.2196/78824 (PMC13427074; doi:10.2196/78824)
Supplement: Multimedia Appendix 3 [file humanfactors-v13-e78824-s003.docx]

**Multimedia Appendix 3.** Stimulus (Phase 2).

**
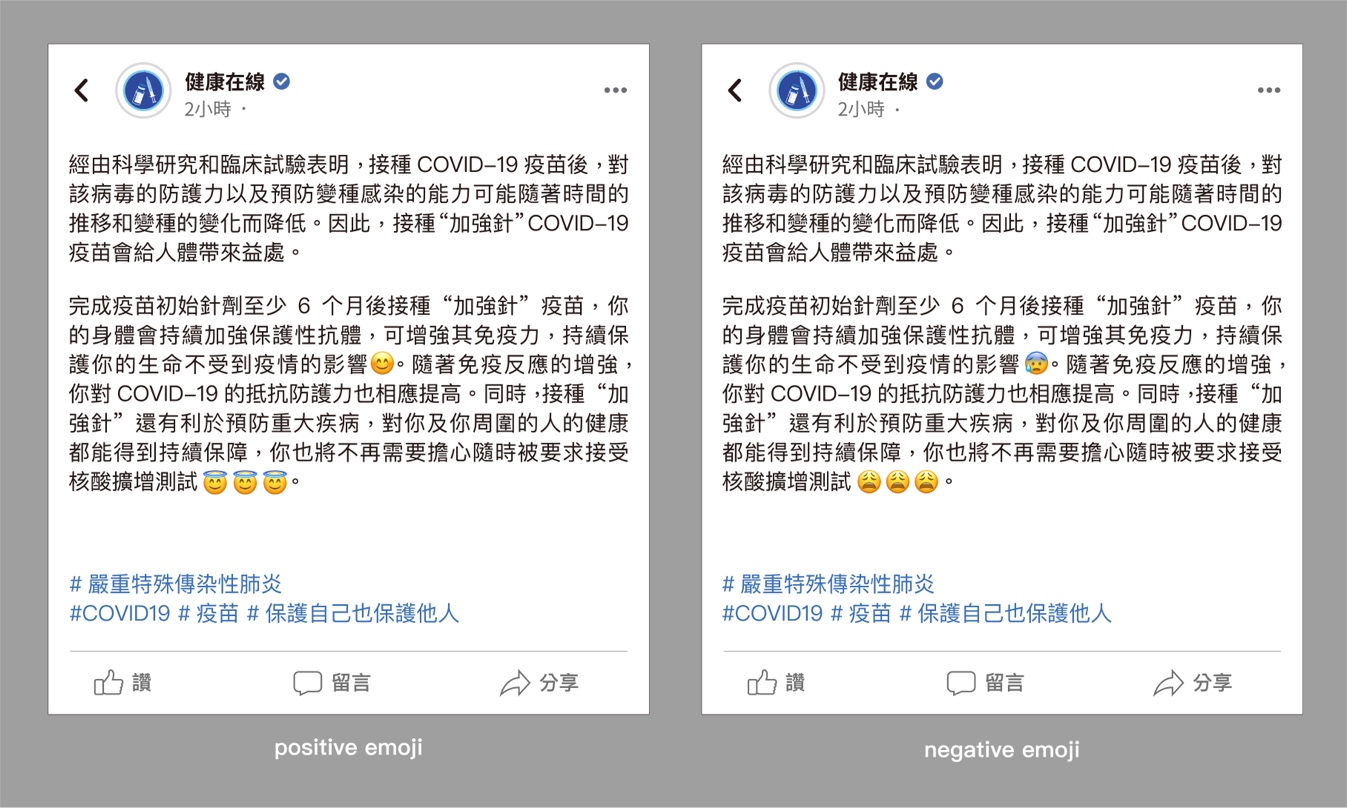
**

Note: The original text is in Traditional Chinese. Please see below the English translation.

According to scientific research and clinical trials, it has been shown that the protective efficacy of the COVID-19 vaccine against the virus and its ability to prevent infections from new variants may decrease over time. Therefore, even after receiving the initial COVID-19 vaccine, it may be beneficial to receive a booster dose.

Receiving a booster shot of the COVID-19 vaccine at least six months after completing the initial dosage can help your body continue to strengthen its protective antibodies, enhance your immunity, and provide continued protection against the impact of the pandemic on your life [emoji]. As your immune response strengthens, your resistance and protective power against COVID-19 will also increase accordingly. Additionally, getting a booster shot can help prevent serious illness and provide continued protection for your health and those around you, eliminating the need to worry about being required to undergo nucleic acid amplification testing at any time [emoji] [emoji] [emoji].
