# Supplementary material for: Strategic Use of Negative Emojis in Messaging-Based Interventions for Public Health Communication on Social Media: Mixed Methods Study
Source: JMIR Hum Factors. 2026 Jul 31;13:e78824. doi: 10.2196/78824 (PMC13427074; doi:10.2196/78824)
Supplement: Multimedia Appendix 4 [file humanfactors-v13-e78824-s004.docx]

**Multimedia Appendix 4.** Stimulus (Phase 3).


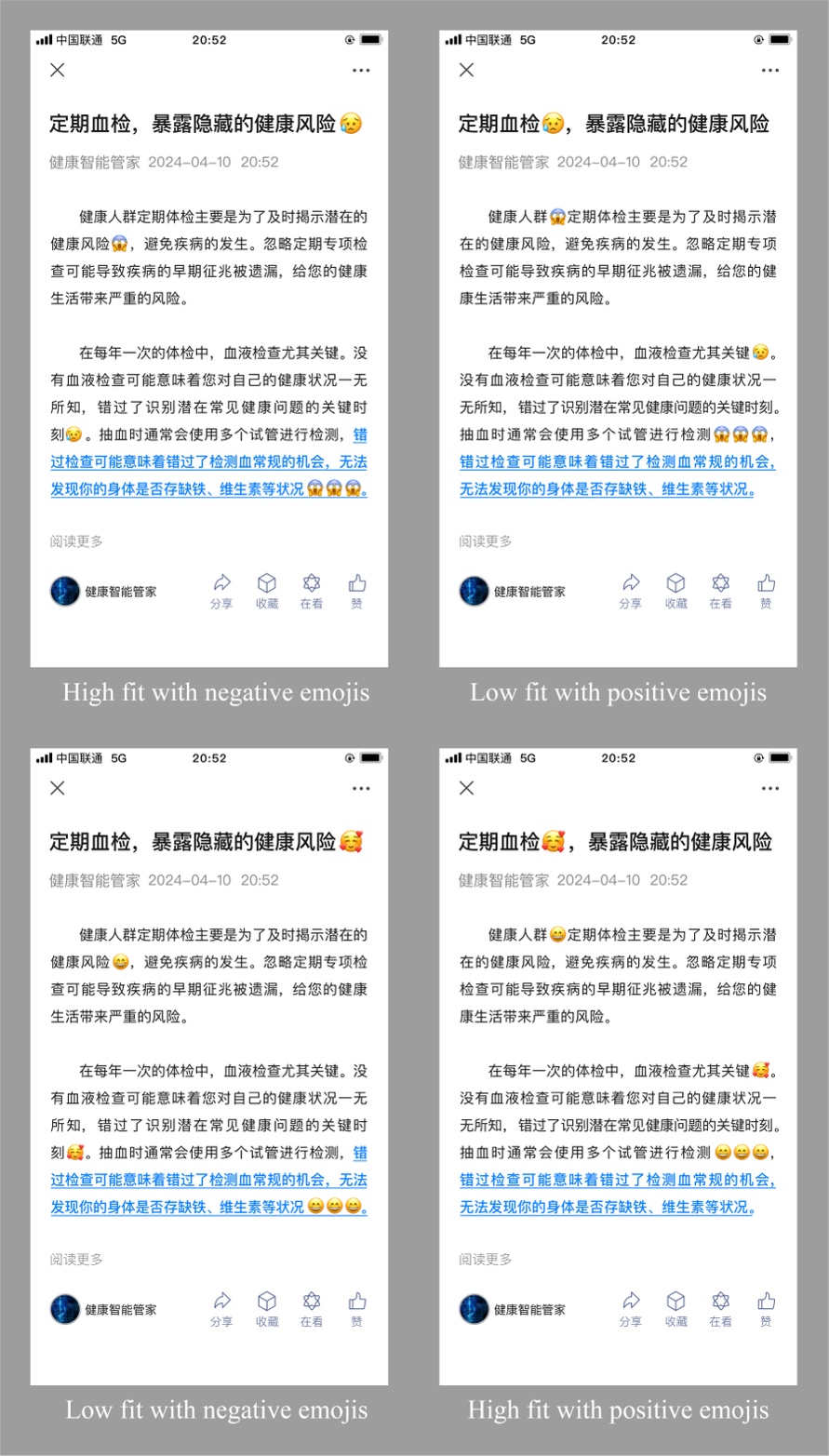


Note: The original text is in Chinese. Please see below the English translation.

| **Congruence** | High (low) fit with negative (positive) emojis | Low (high) fit with negative (positive) emojis |
| --- | --- | --- |
| **Title** | Regular blood tests, expose hidden health risks [emoji] | Regular blood tests [emoji], expose hidden health risks |
| **Content** | The main purpose of regular health check-ups for healthy people is to timely reveal potential health risks [emoji] and prevent the occurrence of diseases. Ignoring regular specialized check-ups may lead to early signs of diseases being overlooked, bringing unnecessary risks to your healthy life.  In the annual health check-up, blood tests are especially crucial. Not having a blood test could mean that you are unaware of your own health condition, missing the key moment to identify potential common health problems [emoji]. During blood collection, multiple tubes are usually used for testing; missing the check-up could mean missing the chance to test your blood count, making it impossible to detect whether your body is lacking in iron, vitamins, etc. [emoji] [emoji] [emoji] | The main purpose of regular health check-ups for healthy people [emoji] is to timely reveal potential health risks and prevent the occurrence of diseases. Ignoring regular specialized check-ups may lead to early signs of diseases being overlooked, bringing unnecessary risks to your healthy life.  In the annual health check-up, blood tests are especially crucial [emoji]. Not having a blood test could mean that you are unaware of your own health condition, missing the key moment to identify potential common health problems. During blood collection, multiple tubes are usually used for testing [emoji] [emoji] [emoji]; missing the check-up could mean missing the chance to test your blood count, making it impossible to detect whether your body is lacking in iron, vitamins, etc. |
